# Heart Rate Variability and Electrocardiographic Parameters Predictive of Arrhythmias in Dogs with Stage IV Chronic Kidney Disease Undergoing Intermittent Haemodialysis

**DOI:** 10.3390/ani10101829

**Published:** 2020-10-08

**Authors:** Angélica Alfonso, André N. V. Le Sueur, Silvano S. Geraldes, Priscylla T. C. Guimarães-Okamoto, Miriam H. Tsunemi, Daniela F. Santana, Victor R. F. Ribeiro, Alessandra Melchert, Simone B. Chiacchio, Maria Lucia G. Lourenço

**Affiliations:** 1 Department of Veterinary Clinic, School of Veterinary Medicine and Animal Science, São Paulo State University (UNESP), Botucatu, 18618-970 São Paulo, Brazil; alfonso_angelica@ymail.com (A.A.); andre.nlsv@gmail.com (A.N.V.L.S.); silvanoport@hotmail.com (S.S.G.); tatiana.okamoto@unesp.br (P.T.C.G.-O.); daniela.santana91@hotmail.com (D.F.S.); mv.victorribeiro@gmail.com (V.R.F.R.); alessandra.melchert@unesp.br (A.M.); sb.chiacchio@unesp.br (S.B.C.); 2 Bioscience Institute, São Paulo State University (UNESP), Botucatu, 18618-970 São Paulo, Brazil; m.tsunemi@unesp.br

**Keywords:** cardiology, electrocardiogram, nephrology, canine

## Abstract

**Simple Summary:**

Monitoring the cardiovascular system plays an important role in this treatment to detect cardiovascular repercussions in dogs with chronic kidney disease (CKD) treated with intermittent haemodialysis (IHD). This study aimed to describe the time-domain and frequency-domain heart rate variability indexes, P and QT dispersions and electrocardiographic alterations observed in dogs with Stage IV CKD undergoing IHD. Animals were divided into three groups, control (10 healthy dogs), clinical treatment (10 dogs with CKD IV submitted to clinical treatment) and IHD (10 dogs with CKD IV submitted to clinical treatment and to dialysis treatment). Clinical, laboratory, HRV indexes and electrocardiographic parameters, as well as QT and P-wave dispersions, were assessed in both CKD groups, prior to and after the end of each clinical treatment/IHD session during the first three sessions. Dogs with CKD IV undergoing IHD had clinically important electrolyte imbalances, electrocardiographic findings, such as the occurrence of arrhythmias and increases in possible predictive parameters for arrhythmias. HRV indexes were better in IHD group, and haemodialysis was more effective at reducing levels of creatinine, urea and phosphorus when compared to intravenous fluid therapy treatment.

**Abstract:**

Intermittent haemodialysis (IHD) is used in dogs with chronic kidney disease (CKD) to reduce azotaemia. Monitoring the cardiovascular system plays an important role in this treatment to detect cardiovascular repercussions. Heart rate variability (HRV) and dispersions of the QT interval and P wave are important markers for mortality risk in humans. This study aimed to describe the time-domain and frequency-domain heart rate variability indexes, P and QT dispersions and electrocardiographic alterations observed in dogs with Stage IV CKD undergoing IHD. Thirty dogs of both sexes, of varying ages and breeds, and weighing between 15 and 30 kg were used. Animals were divided into three groups, control (10 healthy dogs), clinical treatment (10 dogs with CKD IV submitted to clinical treatment twice a week) and IHD (10 dogs with CKD IV submitted to clinical treatment and to dialysis treatment with intermittent haemodialysis twice a week). Clinical, laboratory, HRV indexes and electrocardiographic parameters, as well as QT and P-wave dispersions, were assessed in both CKD groups, prior to and after the end of each clinical treatment/IHD session during the first three sessions. Dogs with CKD IV undergoing IHD had clinically important electrolyte imbalances, primarily hypokalaemia, and pertinent electrocardiographic findings, such as the occurrence of supraventricular arrhythmias and increases in possible predictive parameters for arrhythmias. In spite of these observations, HRV indexes were better in animals undergoing haemodialysis and, in addition, IHD was more effective at reducing levels of creatinine, urea and phosphorus compared to intravenous fluid therapy treatment.

## 1. Introduction

Chronic kidney disease (CKD) is a common diagnosis in small animals and is caused by irreversible morphofunctional injuries in the renal parenchyma, resulting in failures of the regulatory/excretory function of the kidneys [1]. Considering the poor prognosis of CKD patients at stage IV of the disease, new therapeutic options, such as intermittent haemodialysis (IHD) and peritoneal dialysis, are being increasingly applied in cases of acute kidney injury and chronic kidney disease [2]. However, the effects of these procedures on the cardiovascular system of dogs with CKD are unknown.

Several electrocardiographic markers have been studied as tools for the diagnosis and risk stratification of individuals in human medicine with heart diseases. Noteworthy among these markers are dispersion of the QT interval [3] and dispersion of the P wave [4,5].

Dispersion of the QT interval (dQT) reflects regional differences in ventricular repolarization [3]. It is believed that increases in dispersion of the ventricular recovery time indicates higher risk for severe ventricular arrhythmias and sudden death in humans [6]. Dispersion of the P wave (dP) has been used as a prognostic index for the development of atrial fibrillation (AF). This index is defined as the difference between the maximum and minimum durations of the P wave registered on different ECG leads [4,5]. Ozben et al. [7] observed that dispersion of the P wave increased considerably during haemodialysis (HD) sessions.

Heart rate variability (HRV) reflects the antagonistic oscillatory influence of the sympathetic and parasympathetic branches of the autonomic nervous system (ANS) on the sinus node, and its evaluation enables analysis of the response of the ANS in the face of stress and pathological conditions [8]. This index represents an important prognostic marker for chronic diseases and therapeutic efficacy, as reported in humans with CKD undergoing dialysis [9,10].

Electrophysiological monitoring is essential in the setting of renal dysfunction, especially during treatment, to recognise the cardiovascular repercussions early, allowing a better prognosis for the patient. Therefore, the objective of the present study was to describe the effects of IHD over the HRV indexes and electrocardiographic parameters predictive of arrhythmias in dogs with CKD undergoing this type of therapy.

## 2. Animals, Materials and Methods

All experimental procedures were performed after approval by the Ethics Commission on Animal Use of the School of Veterinary Medicine and Animal Science of the São Paulo State University (UNESP), Botucatu, Brazil, under protocol number 61/2016. Animals used in this prospective study were included only following the signing of an informed consent form by the owner.

### 2.1. Animals and Study Design

Twenty client-owned dogs of varying sexes and breeds native to the city of Botucatu and nearby regions, weighing between 15 and 30 kg, were prospectively selected from the patient population of the Nephrology and Urology Small Animal Service of the Teaching Hospital of the School of Veterinary Medicine and Animal Science, São Paulo State University. Additionally, for the control group, ten healthy client-owned dogs were included, with the same weight range as the others.

The selection criteria for all dogs with IRIS CKD Stage 4 was based in a 30-day evaluation period before inclusion to the study according to the staging system defined by the International Renal Interest Society (IRIS) [11]. Weekly laboratory evaluation including CBC, serum chemistry assay, urinalysis, urinary protein: creatinine ratio (UPC), venous blood gas analysis, systolic blood pressure (SBP) by Doppler Vascular (Parks Medical Electronics, Aloha, OR, USA) vector-borne disease PCR (IDEXX Laboratories, Westbrook, ME, USA) were performed when necessary. All patients also had thoracic radiography and abdominal ultrasonography performed.

Dogs that developed acute-on-chronic kidney disease, uremic syndrome, shock, sepsis or were being treated for pancreatitis, autoimmune diseases, congestive heart failure, history of cardiac trauma or cardiopulmonary resuscitation for a period of 90 days before consultation, neoplasia, cardiotoxic medications, coagulation disorders, infectious diseases, nephrolithiasis or had a previous diagnosis of familial or congenital renal or cardiopathy disease were excluded from the study.

Dogs with IRIS CKD Stage 4 were divided into two groups: conventional CKD treatment (CT) (*n* = 10) and IHD (*n* = 10). Patient selection was based on the pet owner’s availability and location of residence. Dogs in the IHD group underwent up to five treatment sessions 2–3 times per week with a 48 h minimum interval. Dogs in the CT group received treatment as needed for CKD either at the teaching hospital or at home from their owners. Dogs in both groups were evaluated every 48 h during the 2-week study. Last, a third group (*n* = 10) of healthy dogs from students, staff, residents and professors, were included as a control group (CG) after all physical and laboratory examinations were performed, as described previously.

### 2.2. Conventional CKD Treatment

Conventional CKD treatment consisted of an individualised stepwise prescription based on physical and laboratory tests to maintain quality of life and longevity in dogs with CKD. Fluid therapy was based on an isotonic polyionic replacement crystalloid, such as lactated Ringer’s solution. Fluid rate was based on estimation of hydration of the patient (body weight × estimated dehydration deficit as a percentage) plus a maintenance rate (2–6 mL/kg/hr) and a volume to account for ongoing loss (polyuria, vomiting, or diarrhoea) if present. During hospital surgery (12 h), a constant rate infusion (CRI) was utilised, but most patients also received SC fluids from their owners at home on days when absent from the study. Furthermore, SBP was monitored at each visit throughout the study period.

All pharmacological treatments were also administered at recommended dosages [12]. Proton pump inhibitors and antiemetics, such as omeprazole (0.5–1 mg/kg PO q 12 h–24 h), ondansetron (0.1 mg/kg PO q 8 h) and maropitant citrate (1 mg/kg SC q 24 h for 5 days), were prescribed for gastroprotection and to control nausea and vomiting if needed. Human recombinant erythropoietin (100 UI/kg SC q 48 h) was used to control non-regenerative anaemia when haematocrit was ≤15%, and iron supplementation was administered as an adjuvant therapy along with erythropoietin treatment [12]. All hypertensive (SBP ≥ 160 mmHg) and proteinuric dogs received an angiotensin-converting-enzyme inhibitor as monotherapy or combined with a calcium channel blocker if needed. In this prospective study, all dogs were proteinuric. Lastly, hyperphosphatemia was managed using aluminium hydroxide (90 mg/kg PO q 24 h) and a commercial renal diet [13].

### 2.3. Intermittent Haemodialysis (IHD)

**Figure 1:**
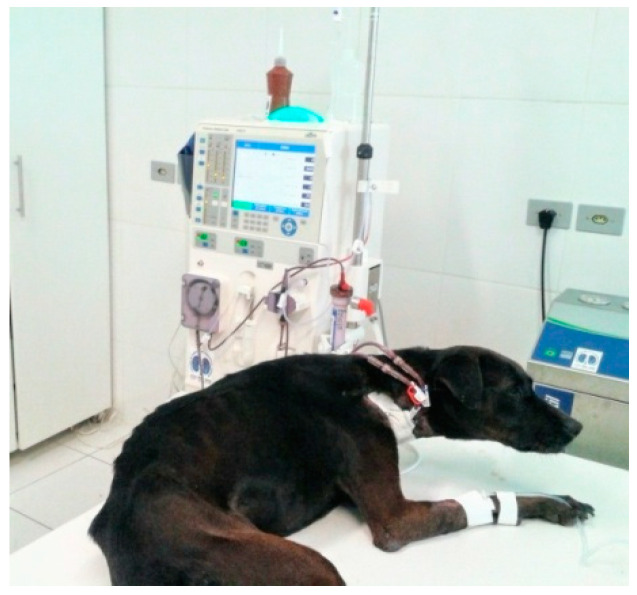
Dog with International Renal Interest Society (IRIS) chronic kidney disease (CKD) Stage 4 during intermittent haemodialysis (IHD) session.

IHD was conducted twice a week with an average duration of 240 to 300 min per session. All dialysis sessions were performed using a 4008S Fresenius machine (Fresenius Medical Care^®^, Waltham, MA, USA) (Figure 1). All dogs were catheterised with an 11 French double-lumen catheter in the right jugular vein using the Seldinger technique [14]. Anticoagulation was ensured using sodium heparin at an initial dosage of 50 U/kg. For dialysis prescription and adequacy, an algorithm based on the urea reduction ratio (URR) was used after treatments. Because of inexperience with dialysis adequacy, an empirical blood flow (Qb) was set according to serum urea concentrations, and haemodialyzers (Hemoflow, Fresenius Medical Care^®^) were chosen according to the patient’s body weight based on veterinary literature [2,15]. All lines and dialyzers received a priming solution of sterile saline.

Prescriptions for IHD were set with a Qb between 2 to 5 mL/kg/min, ultrafiltration (UF) rates were kept constant between 5 and 10 mL/kg/h because of the priming solution of 120 mL and subsequently because of boluses of crystalloids used as a consequence of poor catheter performance in three dogs. A bicarbonate solution (BiBag, Fresenius Medical Care^®^) was added to the dialysate solution and kept at a constant flow rate (Qd) of 500 mL/min during all sessions. An activated clotting time machine kit MCA 2000 (Adib Jatene Fundation) was used to measure the anticoagulant effect hourly, and additional boluses of heparin were administered if necessary. SBP was also monitored by Doppler Vascular (Parks Medical Electronics), following the recommendations for monitoring blood pressure as described by Acierno et al. [16], every 30 min throughout the dialysis treatment.

### 2.4. Clinical and Laboratory Evaluations

In the CT group, blood was drawn via jugular venepuncture at baseline (Pre-CT) and 30 min after fluid therapy treatment (Post-CT) for each in-hospital session. In the IHD group, baseline samples were collected from the double-lumen catheter (Pre-IHD) and 60 min after dialytic therapy (Post-IHD) to avoid a recirculation effect. After collection, blood was transferred into serum tubes and centrifuged at 3000 *g* for 10 min. All assays were performed according to the manufacturers’ recommendations.

Both groups underwent urinalysis by cystocentesis and had their UPC evaluated to assess the degree of proteinuria. In addition, venous blood was also collected for blood gas evaluation in a portable device with the Cg8+ kit at both the aforementioned time points for instant evaluation of ionised calcium, potassium, pH, haematocrit and metabolic acidosis, all of which are essential data for adequation of the haemodialysis procedure.

### 2.5. Electrocardiogram

Electrocardiographic examinations were conducted using a computer-based electrocardiograph (TEB ECG-PC VET^®^, Brazilian Electronic Technology, São Paulo, Brazil) at two distinct time points over three sessions: before and after each session for clinical treatment and IHD. In addition, in the control group, the exam was performed only once for comparison with the treatment groups after the third session.

The examination was conducted without anaesthesia or other type of sedation. Electrodes were secured on the skin at the areas of the humerus-radius-ulna and femur-tibia-patella joints, as standardised by Tilley [17]. Three bipolar leads (I, II and III) and three augmented unipolar leads (aVR, aVL and aVF) were registered.

Electrocardiographic interpretation, in addition to cardiac rhythm, also assessed the following parameters that are predictive for arrhythmias.

Determination of dispersion of the P wave: in each lead evaluated, the duration of the P wave was measured as the distance between its beginning and end, registered in seconds. As such, the minimum (Pmin) and maximum (Pmax) durations of the P wave were determined for each lead. The dispersion of the P wave (Pd) was calculated as the difference between Pmax and Pmin (Pd = Pmax–Pmin). The arithmetic mean was calculated with the measurements of three distinct cardiac cycles.

Determination of the dispersion of the QT interval: in each lead evaluated, the duration of the QT interval was measured as the distance between its beginning and end, registered in seconds. As such, the minimum (QTmin) and maximum (QTmax) durations of the QT interval were determined for each lead. Dispersion of the QT interval (QTd) was calculated as the difference between QTmax and QTmin (QTd = QTmax–Qtmin). The arithmetic mean was calculated using the measurements of three distinct cardiac cycles

### 2.6. Heart Rate Variability (HRV)

ECG tracings underwent HRV analysis using a software (Kubios HRV 3.1, Kubios, Kuopio, Finland). The time-domain and frequency-domain HRV indexes were analysed. The time-domain indexes examined included heart rate (HR), RR interval (distance between two consecutive R waves), standard deviation of the means of normal RR intervals calculated at five-minute intervals (SDNN) and the square root of the mean squared differences of consecutive RR intervals (RMSSD). SDNN was used to reflect all cyclic components of variability in the registered series of RR intervals. RMSSD was used as an estimate of high frequency variations in short-term RR recordings.

The frequency-domain indexes examined included low frequency measured in normalised units (LF nu), high frequency measured in normalized units (HF nu) and the LF/HF ratio. LF was used to examine sympathetic modulations, while HF was used to assess parasympathetic modulations. The LF/HF ratio was used to assess the sympathovagal balance.

### 2.7. Statistical Analysis

Results were analysed using R statistical software (R Core Team 2013, R Foundation for Statistical Computing, Vienna, Austria), and the normality test (Shapiro Wilk) was performed for all parameters. For comparison between groups I (Clinical Treatment) and II (IHD) at each moment (before and after) and at each session (1st, 2nd and 3rd), we employed Levene’s test for equality of variances, the Mann-Whitney test for results with normality > 0, the t1 test for independent samples (equivalent variances) and the t2 test for independent samples (different variances). For comparison between groups after the third session, control versus clinical treatment and control versus IHD, the variables that presented the same normality and variance, ANOVA multiple comparisons test was used, if normality and variance differed, Kruskall Wallis was used. The level of significance considered for these tests was 5% (*p* < 0.05). In addition, for the IHD group, we used an analysis of covariance (ANCOVA) between the electrolytes and arrhythmias.

## 3. Results

The clinical treatment group was comprised of two females and eight males with an average weight of 21.09 ± 8.53 kg and average age of 9.8 ± 2.89 years. Seven dogs were mixed-breeds, two were Border Collies and one was a Rottweiler. The IHD group was comprised of three females and seven males with average weight of 23.05 ± 10.82 kg and average age of 9.9 ± 3.0 years. Five dogs were mixed-breeds, one was an American Pitbull, one was a Weimaraner, one was a Rottweiler, one was a Chow and one was a Labrador Retriever.

**Figure 2:**
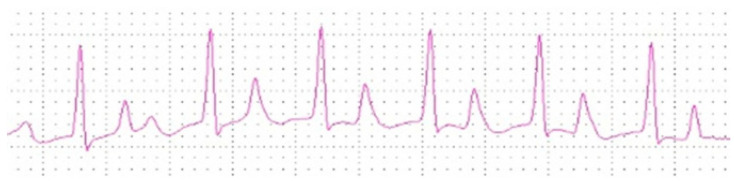
Supraventricular arrhythmia in dog with IRIS CKD Stage 4 after IHD session (25 mm/s; 1 cm = 1 mV).

Regarding the arrhythmias observed, only sinus tachycardia (HR greater than 160 bpm) was obtained after the fluid therapy sessions for dogs in the clinical treatment group in 23% (7/30) of the animals, with sinus arrhythmia in 17% (5/30) and normal sinus rhythm in 60% (18/30). One animal exhibited a first-degree atrioventricular block before and after the three fluid therapy sessions, while another showed isolated premature atrial contraction before the second session. On the other hand, after the IHD sessions, approximately 13% (4/30) of animals showed temporary sinus arrest, 10% (3/30) showed paroxysmal atrial tachycardia (Figure 2), 3% (1/30) showed sustained atrial tachycardia, 17% (5/30) showed sinus tachycardia, 13% (4/30) showed sinus arrhythmia and 43% (13/30) showed sinus rhythm. Two animals showed premature ventricular contractions before IHD sessions, and one animal showed an isolated premature atrial contraction after the second IHD session (Table 1).

The IHD group showed higher values for HR after each treatment session when compared to the clinical treatment group, but at no time point was this increase statistically significant (*p* > 0.05). For the predictive parameters for arrhythmias, after the first session, we noticed higher values for dispersion of the P wave in the IHD group (*p* = 0.057) (Table 2), with the maximum duration of the QT interval (*p* = 0.028) and the dispersion of the QT interval (*p* = 0.043) also higher in this group. In the third session, dispersion of the P wave was significantly higher in the IHD group, both before and after the treatment session (*p* < 0.05).

Table 3 describes the HRV indexes during the three treatment sessions for the clinical treatment and IHD groups. The IHD group exhibited higher values for RMSSD and SDNN after the first session and before the third session compared to the clinical treatment group. In addition, LF was lower after the first IHD session than after the first fluid therapy session. HR, HF and the LF/HF ratio did not present statistically significant differences between the groups during the three treatment sessions (*p* > 0.05).

Table 4 shows the concentrations of electrolytes, urea, creatinine and bicarbonate, as well as pH and SBP, over the sessions for both groups. In general, animals undergoing treatment with IHD exhibited lower concentrations of potassium and higher concentrations of sodium and magnesium, both before and after the three treatment sessions. The IHD group showed higher concentrations of calcium only after the third session. Levels of creatinine and phosphorus were lower after the three haemodialysis sessions, while the inverse was observed for levels of bicarbonate and pH. Concentrations of urea were significantly lower in the IHD group after the second and third sessions.

**Figure 3:**
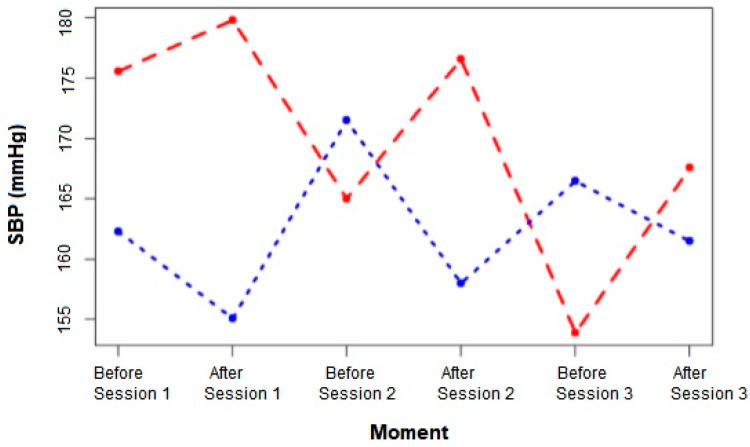
Mean values for SBP in dogs IRIS CKD Stage 4 undergoing CT (red line) and IHD (blue line) after three treatment sessions.

Corpuscular volume (CV), the systolic blood pressure (SBP) and urinary protein-to-creatinine ratio (UPC) did not diverge significantly between the groups either before or after each session. Although not statistically significant, SBP was progressively lower with each session in both groups (Table 4). Animals undergoing clinical treatment started the first session with a mean SBP of 175.0 ± 27.75 mmHg and finished the third session with 167.0 ± 31.66 mmHg, while animals undergoing IHD started the first session with 162.0 ± 35.53 mmHg and finished the third session with 161.0 ± 26.04 mmHg. In addition, animals in the clinical treatment group presented higher SBP after each treatment than they did before, while animals in the IHD group showed lower SBP after each treatment than they did before (Figure 3).

Table 5 describes the predictive parameters for arrhythmias and HRV indexes in the groups submitted to clinical treatment and IHD obtained after the third session and compared to data obtained from healthy animals (control group). In both groups (clinical treatment and IHD), duration of the maximum P wave, dispersion of the P wave and dispersion of the QT interval were longer than in the control group, and animals submitted to clinical treatment obtained a longer duration of the minimum P wave compared to the control group. HRV indexes did not differ among the three groups, however, the time domain indexes were higher in the IHD group than in the group submitted only to fluid therapy, whereas the LF/HF ratio and LF were higher in the IHD group.

SBP was not correlated with any HRV index over the first two treatment sessions for either group, but at the third session, the IHD group exhibited correlations of this parameter with high frequency (HF) (before: ρ = −0.65; *p* = 0.04; after: ρ = −0.79; *p* = 0.006), low frequency (LF) (before: absent; after: ρ = 0.79; *p* = 0.006) and LF/HF ratio (before: ρ = 0.62; *p* = 0.05; after: ρ = 0.81; *p* = 0.003). When ANCOVA was applied, we observed a negative correlation in the IHD group between electrolytes and the presence of arrhythmias (correlation coefficient = −1.81; *p* = 0.021) and between serum concentrations of potassium and the presence of arrhythmias after all three IHD sessions.

## 4. Discussion

This study revealed that dogs with CKD undergoing treatment with IHD exhibited important alterations in concentrations of electrolytes and in resting ECG, such as the occurrence of supraventricular arrhythmias and increases in the possible predictive parameters for arrhythmias. In spite of these observations, HRV indexes were better in animals undergoing haemodialysis and, in addition, IHD was more efficient in reducing levels of creatinine, urea and phosphorus compared to intravenous fluid therapy treatment.

In the CT group, although dispersions of the P wave and QT interval were higher than in controls, we did not observe any severe ventricular or supraventricular arrhythmic events related to the increase in these dispersions. Sinus tachycardia was observed during all three treatment sessions, as were electrolytic alterations, such as hyperphosphatemia and hypocalcaemia, which are common in dogs suffering from CKD [1]. Sinus tachycardia has been described as an expected consequence in dogs with hypocalcaemia because the excitability of the membrane is influenced by the concentration of ionised calcium [17]. Hypocalcaemia is a recognised cause of extended QT intervals because it extends the plateau phase of the cardiac action potential, which keeps the ionic channels for calcium open longer, allowing a late influx of calcium and early depolarisations. When the depolarization threshold is reached, new action potentials are induced, inducing tachycardia [18].

As for the arrhythmias detected, approximately 42% (14/30) of dogs in the study developed some kind of rhythm alteration after the IHD sessions. The reduction of blood volume over a short time span, electrolytic and metabolic alterations, alterations in the acid-base balance, reductions in the effective circulatory volume and the high prevalence of risk factors for the development of cardiovascular abnormalities were factors that contributed toward this higher occurrence of arrhythmic events during dialysis [19].

Dispersion of the P wave in the IHD group over the three treatment sessions was higher than in the clinical treatment group, with important supraventricular arrhythmic events observed at three of the six time points examined. In addition to hypocalcaemia and hyperphosphatemia (also observed in the fluid therapy group), animals in the IHD group also presented with hypokalaemia and hypomagnesemia. Animals in the present study were hypokalaemic before the first session, likely because of the loss of electrolyte resulting from chronic kidney disease [1], and this low potassium rate was maintained by the loss of electrolytes during the dialysis process [2]. Potassium replacement was performed throughout the treatment, however, because of the absence of 24-h hospitalization in our veterinary hospital unit, this replacement was not always effective because of the short stay of the animal throughout the day, and replacement of this electrolyte must be done slowly.

Negative correlations between arrhythmias and serum concentrations of potassium were observed after the haemodialysis treatment sessions, indicating through analysis of covariance that these events may have been caused not by the IHD process, but by the hypokalaemia. The most likely mechanism through which potassium leads to a higher risk of supraventricular arrhythmias is the influence of this electrolyte on the potential of cell membranes. It is believed that low levels of potassium may cause cellular hyperpolarization, increase the resting potential and accelerating myocardial depolarization [20].

Regarding the long QT interval in dogs, Ware et al. [21] related prolongation of the QT interval and sudden death in dogs of the English Springer Spaniel breed, and Brüler et al. [22] concluded that QT interval prolongation and instability were significantly related to mortality in dogs and may be useful predictors in the prognosis of patients with myxomatous valve disease. In our study, no sudden death occurred, but the maximum duration of the QT interval and the dispersion of the QT interval were significantly higher in the IHD group immediately after the first session compared to the clinical treatment group, and significant differences in QT dispersion were observed when compared to the control group at the end of the third treatment session. Yetkin et al. [23] observed a significant correlation between increases in the dispersion of the QT interval and the degree of electrolytic alterations after haemodialysis sessions. Even though dialysis normalises serum electrolytes, sudden changes in the concentrations of potassium and calcium or in the pH may contribute to increases in the dispersion of the QT interval. Potassium and magnesium are two important factors for electrical stability of the myocardium, and both are involved in the creation of cellular excitability, the propagation of impulses and in regular ventricular recovery [24].

In this study, we observed higher values for the maximum duration of the P wave and for dispersion of the P wave after the third haemodialysis session compared to the clinical treatment group and with the control group. Therefore, after the IHD session, there was an increase in the conduction time and a dispersion of the atrial refractiveness, which may induce atrial fibrillation, in particular after the sessions. Korzets et al. [25] verified that when human patients undergoing dialysis reached a potassium concentration of 2 mEg/L, they became hypokalaemic during the last half of a standard 4-h treatment, the time during which atrial fibrillation commonly occurs. Hypokalaemia is considered a risk factor both for ventricular and supraventricular arrhythmias [26].

In parallel, we observed significantly lower serum concentrations of potassium after the dialysis sessions compared to the clinical treatment group. A study conducted by Tezcan et al. [27] also observed a significant reduction in potassium concentrations after dialysis, and this reduction was negatively correlated with the values obtained for the maximum duration of the P wave and dispersion of the P wave. Korzets et al. [25] recommended dialysis for human patients with potassium concentrations of 3 mEg/K when a history of atrial fibrillation is detected during the sessions.

Szabo et al. [28] also examined the effects of haemodialysis on the duration and dispersion of the P wave in humans. They discovered that the maximum duration and dispersion of the P wave significantly increased after each haemodialysis session. However, when they divided patients according to the diameter of the left atrium, dispersion of the P wave significantly increased after haemodialysis only in patients with diameters of the left atrium exceeding 45 mm, indicating that haemodialysis treatment in association with larger left atriums increase the chances of atrial fibrillation occurring.

In spite of the electrolytic alterations, arrhythmias and altered predictive parameters observed in the IHD group, we observed that HRV indexes were lower in that group compared to animals treated only with conventional fluid therapy. After the third session, the HRV indexes for both groups did not differ from the control group. After the first and third treatment sessions, RMSSD and SDNN were significantly higher in the IHD group than in the clinical treatment group, and immediately after the first session, the low frequency (LF) component was lower in dogs undergoing haemodialysis than in dogs undergoing only conventional clinical treatment. Studies regarding the effects of renal replacement therapy with respect to HRV index are still highly controversial in human medicine and practically non-existent in veterinary medicine.

In a longitudinal study, Mylanopolou et al. [29] observed a significant increase in SDNN values in patients undergoing haemodialysis, while the frequency-domain indexes were not altered. Giordano et al. [30] observed an improvement in autonomic neuropathy in patients undergoing haemodialysis. In contrast, Axelrod et al. [31] studied eight patients treated with haemodialysis, seven with peritoneal dialysis and ten with conservative treatment, observing a decrease in autonomic control in patients undergoing haemodialysis and peritoneal dialysis, while patients undergoing conservative treatment presented attenuated decreases.

One study conducted in humans demonstrated that haemodynamic instability during haemodialysis is strongly related to decreases in HRV (particularly of SDANN) and to the behaviour of sympathovagal balance (lower LF/HF), suggesting compromised autonomic control in uremic patients [32]. Intradialytic hypotension is described as a common occurrence both in veterinary medicine [11] and in human patients undergoing this type of treatment and is considered a manifestation of autonomic nervous system dysfunction, leading to an insufficient sympathetic response to ultrafiltration induced by the hypovolemia [33]. In this study, we did no observe hypotension after the IHD sessions, which may have contributed to the maintenance of improved HRV indexes in that group in association with other factors, such as a significant decrease after sessions in levels of urea, creatinine, phosphorus and other metabolic agents that interfere in the regulation of heart rate and were removed by haemodialysis, resulting in improved function of autonomic cardiac control in uremic patients [34].

Despite not observing statistically significant differences in SAP between the groups, we observed a decrease in this parameter in both groups as the sessions progressed. Animals in both groups started the treatments with high blood pressures and moderate risk of injury in the target organ [16]. This gradual decrease can be explained by the efficacy of the antihypertensive treatment. We observed that animals in the clinical treatment group always presented with higher systemic arterial blood pressure after treatment compared to before, and animals in the IHD group always presented with lower systemic arterial blood pressures after each session compared to before. This behaviour can be explained by the increased blood volume caused by fluid therapy and by the decreased blood volume caused by IHD treatment [11,14]. In addition, after the third IHD session, the high frequency (HF) index presented a negative correlation with SBP, while the low frequency (LF) index presented a positive correlation with SBP.

The limiting factors for this study include obtaining the HRV data immediately after the sessions; further studies, including a 24 h Holter examination to compare the HRV indexes with values obtained in an ambulatory ECG, may help confirm or deny the behaviour observed in the autonomic nervous system. In addition, each drug in the treatment of both groups varied with the individual needs of each animal, representing a bias in the study.

## 5. Conclusions

Dogs with IRIS CKD Stage 4 undergoing IHD presented with a high rate of abnormal electrocardiographic findings, including arrhythmias and supraventricular arrhythmias, as well as higher values for dispersions of the P wave and QT interval compared to animals submitted only to conventional clinical treatment and to healthy animals.

They also presented more marked electrolyte imbalances after sessions (particularly hypokalaemia and hypomagnesemia) than animals undergoing only conventional clinical treatment. However, dogs undergoing dialysis presented higher HRV indexes than those undergoing only the clinical treatment. In addition, IHD improved the general blood biochemistry by reducing serum concentrations of urea, creatinine and phosphorus more efficiently than the intravenous fluid therapy, leading to improved clinical profiles in these patients.

## Figures and Tables

**Table 1:** Main arrhythmias observed in dogs with International Renal Interest Society (IRIS) chronic kidney disease (CKD) Stage 4 undergoing clinical treatment (CT) and intermittent haemodialysis (IHD) after three treatment sessions.

		1st Session		2nd Session		3rd Session	
Arrhythmias		CT	IHD	CT	IHD	CT	IHD
ST	Before	*n* = 2	*n* = 0	*n* = 1	*n* = 0	*n* = 3	*n* = 2
	After	*n* = 2	*n* = 0	*n* = 2	*n* = 2	*n* = 33	*n* = 2
1st Degree AVB	Before	*n* = 1	*n* = 1	*n* = 1	*n* = 1	*n* = 1	*n* = 1
	After	*n* = 1	*n* = 1	*n* = 1	*n* = 1	*n* = 1	*n* = 1
Isolated APC	Before	*n* = 0	*n* = 0	*n* = 1	*n* = 0	*n* = 0	*n* = 0
	After	*n* = 0	*n* = 0	*n* = 0	*n* = 1	*n* = 0	*n* = 0
Sinus arrest	Before	*n* = 0	*n* = 0	*n* = 0	*n* = 1	*n* = 0	*n* = 0
	After	*n* = 0	*n* = 1	*n* = 0	*n* = 3	*n* = 0	*n* = 0
Paroxysmal AT	Before	*n* = 0	*n* = 0	*n* = 0	*n* = 0	*n* = 0	*n* = 1
	After	*n* = 0	*n* = 0	*n* = 0	*n* = 2	*n* = 0	*n* = 1
Sustained AT	Before	*n* = 0	*n* = 0	*n* = 0	*n* = 0	*n* = 0	*n* = 0
	After	*n* = 0	*n* = 0	*n* = 0	*n* = 0	*n* = 0	*n* = 1
Isolated VPC	Before	*n* = 0	*n* = 1	*n* = 0	*n* = 0	*n* = 0	*n* = 1
	After	*n* = 0	*n* = 0	*n* = 0	*n* = 0	*n* = 0	*n* = 0

ST: sinus tachycardia; AVB: atrioventricular block; APC: atrial premature contraction; AT: atrial tachycardia; VPC: ventricular premature contraction.

**Table 2:** Means and standard deviations of the heart rate (HR) and electrocardiographic parameters possible predictive of arrhythmias (P wave maximum, P wave minimum, dispersion of P wave, QT interval maximum, QT interval minimum, dispersion of QT interval) in dogs with IRIS CKD Stage 4 undergoing CT and IHD, before and after three treatment sessions.

		1st Session		2nd Session		3rd Session	
Parameters		CT	IHD	CT	IHD	CT	IHD
HR (bpm)	Before	135.0 ± 33.69	119.0 ± 20.85	128.0 ± 24.51	120.0 ± 26.14	142.0 ± 30.34	133.0 ± 24.37
	After	129.0 ± 33.38	124.0 ± 24.30	128.0 ± 28.20	138.0 ± 31.65	141.0 ± 30.89	140.0 ± 36.83
Pmax (s)	Before	0.059 ± 0.005	0.059 ± 0.005	0.059 ± 0.007	0.058 ± 0.007	0.058 ± 0.007	0.059 ± 0.007
	After	0.06 ± 0.007	0.062 ± 0.007	0.058 ± 0.008	0.058 ± 0.008	0.06 ± 0.007	0.062 ± 0.007
Pmin (s)	Before	0.046 ± 0.006	0.044 ± 0.006	0.048 ± 0.006	0.042 ± 0.006	0.047 ± 0.007	0.043 ± 0.007
	After	0.05 ± 0.006	0.049 ± 0.006	0.046 ± 0.006	0.043 ± 0.006	0.049 ± 0.006	0.044 ± 0.006
dP (s)	Before	0.014 ± 0.009	0.014 ± 0.004	0.011 ± 0.006	0.015 ± 0.008	0.011 ± 0.006 ^a*^	0.016 ± 0.005 ^b*^
	After	0.01 ± 0.003 ^a*^	0.013 ± 0.004 ^b*^	0.012 ± 0.009	0.015 ± 0.005	0.011 ± 0.006 ^a*^	0.018 ± 0.007 ^b*^
QTmax (s)	Before	0.213 ± 0.021	0.224 ± 0.017	0.213 ± 0.018	0.21 ± 0.019	0.207 ± 0.024	0.2 ± 0.014
	After	0.212 ± 0.020 ^a**^	0.233 ± 0.018 ^b**^	0.214 ± 0.019	0.212 ± 0.021	0.212 ± 0.026	0.202 ± 0.019
QTmin (s)	Before	0.198 ± 0.020	0.208 ± 0.017	0.199 ± 0.021	0.191 ± 0.017	0.19 ± 0.024	0.182 ± 0.012
	After	0.194 ± 0.022	0.209 ± 0.019	0.196 ± 0.020	0.189 ± 0.025	0.195 ± 0.028	0.184 ± 0.020
dQT (s)	Before	0.015 ± 0.005	0.016 ± 0.005	0.014 ± 0.007	0.019 ± 0.007	0.017 ± 0.006	0.018 ± 0.008
	After	0.018 ± 0.013 ^a*^	0.024 ± 0.009 ^b*^	0.017 ± 0.008	0.02 ± 0.005	0.017 ± 0.008	0.017 ± 0.005

bpm: beats per minute; s: seconds. Within each line, different superscripted letters (a, b) mean statistically significant differences (*p* < 0.05). * Mann-Whitney test, ** t1 test.

**Table 3:** Means and standard deviations for the heart rate variability (HRV) indexes (RR interval - RR, standard deviation of the means of normal RR intervals calculated at five-minute intervals – SDNN, square root of the mean squared differences of consecutive RR intervals – RMSSD, low frequency – LF, high frequency – HF, and low frequency/high frequency ratio – LF/HF) of dogs with IRIS CKD Stage 4 undergoing CT and IHD, before and after the treatment sessions.

		1st Session		2nd Session		3rd Session	
Parameters		CT	IHD	CT	IHD	CT	IHD
HR (bpm)	Before	135.0 ± 37.5	120.7 ± 32.23	139.7 ± 33.25	133.3 ± 32.39	135.3 ± 22.63	132.5 ± 28.95
	After	140.0 ± 35.95	115.5 ± 34.25	136.0 ± 29.26	138.7 ± 37.30	137.9 ± 36.43	146.3 ± 28.26
RR (ms)	Before	470.2 ± 112.2	528.8 ± 141.7	452.1 ± 117.18	475.0 ± 124.78	453.3 ± 76.0	474.0 ± 119.94
	After	451.6 ± 108.24	567.1 ± 191.4	454.5 ± 78.72	466.6 ± 147.38	465.8 ± 137.15	422.4 ± 81.98
RMSSD (ms)	Before	42.81 ± 39.6	78.41 ± 65.31	22.86 ± 10.51	85.41 ± 65.82	25.46 ± 16.23 ^a*^	67.99 ± 41.06 ^b*^
	After	27.18 ± 16.32 ^a*^	119.7 ± 60.28 ^b*^	31.51 ± 14.80	85.51 ± 78.33	40.63 ± 24.77	42.14 ± 33.45
SDNN (ms)	Before	39.21 ± 34.78	57.61 ± 0.5	19.53 ± 9.64	65.71 ± 54.91	20.93 ± 14.21 ^a**^	66.52 ± 48.26 ^b**^
	After	22.83 ± 11.23 ^a*^	90.52 ± 76.98 ^b*^	30.52 ± 15.64	76.46 ± 53.42	29.38 ± 22.15	38.28 ± 31.85
LF (n.u.)	Before	30.3 ± 17.25	22.65 ± 15.48	35.68 ± 22.02	32.11 ± 26.44	31.2 ± 17.28	32.13 ± 20.12
	After	38.0 ± 18.75 ^b**^	22.64 ± 11.74 ^a**^	28.25 ± 13.52	35.75 ± 20.88	29.55 ± 16.59	32.64 ± 21.36
HF (n.u.)	Before	69.27 ± 17.21	76.99 ± 15.41	63.68 ± 21.6	67.46 ± 26.2	68.22 ± 17.14	67.51 ± 19.86
	After	63.33 ± 18.66	73.99 ± 21.26	71.33 ± 23.52	64.03 ± 20.94	69.73 ± 16.36	66.81 ± 21.09
LF/HF	Before	0.53 ± 0.49	0.35 ± 0.34	0.75 ± 0.65	0.76 ± 0.66	0.54 ± 0.41	0.6 ± 0.5
	After	1.12 ± 1.02	0.41 ± 0.40	0.6 ± 0.50	0.73 ± 0.63	0.48 ± 0.32	0.64 ± 0.57

Within each line, different superscripted letters (a, b) mean statistically significant differences (*p* < 0.05). * Mann-Whitney test, ** t1 test.

**Table 4:** Means and standard deviations for the laboratory test results and systolic blood pressure (SBP) in dogs with IRIS CKD Stage 4 undergoing CT and IHD, before and after the treatment sessions.

		1st Session		2nd Session		3rd Session	
Parameters		CT	IHD	CT	IHD	CT	IHD
K (mEq/L)	Before	4.3 ± 0.74 ^b**^	2.42 ± 0.69 ^a**^	4.32 ± 0.5 ^b*^	2.52 ± 0.57 ^a*^	4.54 ± 0.38 ^b**^	2.8 ± 0.86 ^a**^
	After	4.27 ± 0.74 ^b**^	2.08 ± 0.42 ^a**^	4.46 ± 0.44 ^b**^	2.14 ± 0.4 ^a**^	4.42 ± 0.73 ^b**^	2.25 ± 0.47 ^a**^
Na (mEq/L)	Before	143.8 ± 2.34 ^a***^	145.7 ± 5.0 ^b***^	143.4 ± 1.07 ^a**^	146.3 ± 4.22 ^b**^	143.7 ± 1.82 ^a*^	145.4 ± 3.68 ^b*^
	After	143.9 ± 3.34 ^a***^	146.0 ± 2.58 ^b***^	143.8 ± 1.62 ^a***^	146.2 ± 3.12 ^b***^	143.6 ± 2.06 ^a*^	145.9 ± 2.55 ^b*^
Mg (mg/dL)	Before	2.94 ± 2.21 ^a*^	3.73 ± 0.61 ^b*^	1.81 ± 0.52 ^a***^	3.57 ± 0.63 ^b***^	1.85 ± 0.64 ^a***^	4.46 ± 1.08 ^b***^
	After	2.38 ± 1.99 ^a*^	3.04 ± 0.48 ^b*^	1.64 ± 0.45 ^a***^	3.13 ± 0.36 ^b***^	1.79 ± 0.70 ^a**^	3.69 ± 1.21 ^b**^
iCa (mmol/L)	Before	0.7 ± 0.15	0.79 ± 0.23	0.7 ± 0.15	0.82 ± 0.28	0.74 ± 0.15	0.83 ± 0.15
	After	0.73 ± 0.15	0.72 ± 0.15	0.7 ± 0.16	0.84 ± 0.17	0.62 ± 0.11 ^a**^	0.83 ± 0.15 ^b**^
P (mg/dL)	Before	13.91 ± 5.68	23.3 ± 22.6	12.32 ± 4.9	8.95 ± 3.66	13.9 ± 5.31	11.39 ± 3.44
	After	12.2 ± 3.34 ^b*^	9.28 ± 9.2 ^a*^	12.29 ± 5.14 ^b*^	5.84 ± 2.69 ^a*^	12.86 ± 5.12 ^b***^	5.37 ± 1.38 ^a***^
Cr (mg/dL)	Before	8.16 ± 3.17	8.07 ± 3.7	8.46 ± 3.16	6.97 ± 2.63	8.75 ± 3.65	7.13 ± 2.13
	After	7.9 ± 3.12 ^b**^	4.44 ± 1.66 ^a**^	7.82 ± 2.87 ^b**^	3.89 ± 1.86 ^a**^	8.07 ± 3.67 ^b**^	3.35 ± 1.79 ^a**^
Urea (mg/dL)	Before	260.0 ± 71.14	313.6 ± 127.78	259.2 ± 63.73	229.4 ± 108.68	264.0 ± 80.61	234.0 ± 76.04
	After	255.8 ± 73.15	191.0 ± 85.95	261.6 ± 77.45 ^b*^	126.0 ± 58.63 ^a*^	255.5 ± 69.15 ^b**^	112.5 ± 52.35 ^a**^
HCO_3_ (mg/dL)	Before	14.9 ± 1.69	16.89 ± 5.44	16.43 ± 2.92	18.84 ± 4.68	16.18 ± 2.15	17.97 ± 3.04
	After	16.34 ± 1.49 ^a***^	21.62 ± 4.35 ^b***^	17.03 ± 1.49 ^a***^	23.74 ± 4.13 ^b***^	16.36 ± 2.27 ^a**^	24.52 ± 4.55 ^b**^
pH	Before	7.29 ± 0.04	7.31 ± 0.1	7.32 ± 0.04	7.35 ± 0.7	7.3 ± 0.04	7.34 ± 0.062
	After	7.31 ± 0.04 ^a**^	7.39 ± 0.08 ^a**^	7.33 ± 0.04 ^a**^	7.42 ± 0.077 ^b**^	7.32 ± 0.04 ^a**^	7.4 ± 0.067 ^b**^
SBP (mmHg)	Before	175.0 ± 27.75	162.0 ± 35.53	165.0 ± 24.71	171.0 ± 25.82	154.0 ± 34.58	166.0 ± 24.04
	After	180 ± 29.0	155.0 ± 29.21	176.0 ± 25.0	158.0 ± 31.10	167.0 ± 31.66	161.0 ± 26.04

Within each line, different superscripted letters (a, b) mean statistically significant differences (*p* < 0.05). * Mann-Whitney test, ** t1 test, *** t2 test.

**Table 5:** Mean and standard deviation of the predictive parameters of arrhythmias and HRV indexes of the control group and of dogs with IRIS CKD Stage 4 after the third session of CT and IHD.

Parameters	Control	CT	IHD
HR (bpm)	131.0 ± 26.38	141 ± 30.89	140.0 ± 36.83
P max (s)	0.044 ± 0.005 ^a*^	0.06 ± 0.007 ^b*^	0.062 ± 0.007 ^b*^
P min (s)	0.04 ± 0.005 ^a**^	0.049 ± 0.006 ^b**^	0.044 ± 0.006
dP (s)	0.004 ± 0.001 ^a**^	0.011 ± 0.006 ^b**^	0.018 ± 0.007 ^b**^
QT max (s)	0.192 ± 0.033	0.212 ± 0.026	0.202 ± 0.019
QT min (s)	0.188 ± 0.033	0.195 ± 0.028	0.184 ± 0.020
dQT (s)	0.004 ± 0.002 ^a**^	0.017 ± 0.008 ^b**^	0.017 ± 0.005 ^b**^
RR (ms)	463.9 ± 94.4	465.8 ± 137.15	422.4 ± 81.98
RMSSD (ms)	74.05 ± 52.86	40.63 ± 24.77	42.14 ± 33.45
SDNN (ms)	58.22 ± 44.46	29.38 ± 22.15	38.28 ± 31.85
LF (nu)	19.69 ± 14.49	29.55 ± 16.59	32.64 ± 21.36
HF (nu)	79.52 ± 14.08	69.73 ± 16.36	66.81 ± 21.09
LF/HR	0.28 ± 0.25	0.48 ± 0.32	0.64 ± 0.57

Within each line, different superscript letters (a, b) denote significant differences (*p* < 0.05). * ANOVA; ** *Kruskall-Wallis*.
